# Back in Action: High Return to Pre-Injury Level of Sports after Arthroscopic Bone Marrow Stimulation for Osteochondral Lesions of the First Metatarsophalangeal (MTP-1) Joint

**DOI:** 10.1177/19476035231200332

**Published:** 2023-09-21

**Authors:** Carlijn S. ter Laak Bolk, Quinten G.H. Rikken, Jari Dahmen, Yoshiharu Shimozono, Masato Takao, Sjoerd A.S. Stufkens, Gino M.M.J. Kerkhoffs

**Affiliations:** 1 Department of Orthopedic Surgery and Sports Medicine, Amsterdam University Medical Center, University of Amsterdam, Amsterdam, The Netherlands; 2 Program Sports and Musculoskeletal Health, Amsterdam Movement Sciences, Amsterdam, The Netherlands; 3 Academic Center for Evidence-Based Sports Medicine, Amsterdam, The Netherlands; 4 Amsterdam Collaboration on Health & Safety in Sports, International Olympic Committee Research Centers, Amsterdam, The Netherlands; 5 Department of Orthopaedic Surgery, Kyoto Shimogamo Hospital, Kyoto, Japan; 6 Clinical and Research Institute for Foot and Ankle Surgery, Jujo Hospital, Kisarazu, Japan

**Keywords:** osteochondral lesions, first metatarsophalangeal joint, clinical, radiological

## Abstract

**Objective:**

The primary aim was to assess the return to sports outcomes of patients with symptomatic osteochondral lesions (OCLs) to the first metatarsophalangeal (MTP-1) joint treated by arthroscopic bone marrow stimulation (BMS). Secondary aims were to present patient-reported outcome measures (PROMs) on pain scores as well as surgery-related complications or reoperations to the MTP-1 joint.

**Design:**

All patients with MTP-1 OCLs treated by arthroscopic BMS with a minimum follow-up of 12 months were included. Outcomes included return to sports and work outcomes, satisfaction outcomes with the performed treatment, PROMs, as well as postoperative complications and reoperations. Medical records were screened by 2 independent reviewers and patients were contacted by phone to partake in an in-depth interview. Complications, reoperations, and revision surgeries were additionally assessed.

**Results:**

Nine patients (median age: 22 years with interquartile range (IQR) 20-29 years) were included with a median follow-up time of 47 (IQR: 23-92) months. Six (86%) out of 7 patients who participated in sports preoperatively returned to sports at any level at a median of 4 (IQR: 2.6-5.8) months. Five patients (71%) returned to pre-injury level of sport and eventually returned to performance at a median of 4 (IQR 2.8-7.5) and 8 (IQR: 4.0-10.5) months, respectively. The median Numeric Rating Scale for pain during walking was 1 (IQR 0-2.5) and all (100%) patients were able to return to work at a median of 4 (IQR: 2-17) weeks. Eighty-nine percent of the patients were very or fairly satisfied with the result of their treatment. No complications, reoperations, or revision surgeries were reported.

**Conclusions:**

Arthroscopic BMS for patients with symptomatic OCLs to the MTP-1 joint can be considered safe and yields an 86% return to sport at any level and a 71% return to pre-injury and performance level, with good clinical, return to work, as well as satisfaction outcomes.

## Introduction

The first metatarsophalangeal (MTP-1) joint is rarely affected by osteochondral lesions (OCLs), as they are mainly found in the elbow, ankle, or knee.^1-5^ OCLs of the first metatarsal head are mainly associated with traumatic events, which may subside from joint compression or direct impact to the metatarsal head.^6-10^ Patients can present with pain, tenderness, swelling, locking, or clicking of the MTP-1 joint. These complaints may result in limitations in activities of daily living or limitations in sports.

Treatment for OCLs of the MTP-1 joint can be performed in a conservative fashion including rest, physiotherapy, injection therapy, weight loss in case of a high body mass index (BMI), as well as casting therapy (partial or full immobilization). However, when conservative treatment fails, and persisting symptoms in daily life continue to hinder the patient, operative options can be considered.^5,11,12^ When assessing the literature, evidence on the efficacy of the different treatments can be considered scarce when regarding the return to sports and clinical outcomes.^5,13-16^ As a result, there is no clear vision on what the return to sports rates, clinical outcomes, and safety outcomes are after different types of treatment of MTP-1 joint OCLs. Finally, the operative technique for arthroscopic approach for MTP-1 (osteo)chondral lesions from the perspective of an expert center in treatment of cartilage injuries in the foot and ankle will be described.

Our primary aim with the present article was to analyze the return to sports outcomes of a selected cohort of patients with symptomatic OCLs to the MTP-1 joint treated by arthroscopic bone marrow stimulation (BMS). Secondary aim was to assess patient-reported outcome measures (PROMs) on pain scores as well as surgery-related complications or reoperations to the MTP-1 joint.

## Materials and Methods

This study included a single-institution cross-sectional follow-up of surgically treated OCLs of the MTP-1 joint at the Department of Orthopaedic Surgery and Sports Medicine from the Amsterdam UMC, the Netherlands, and an in-depth description of arthroscopic approaches from the perspective of a multicenter experience in the treatment of cartilage injuries in the foot and ankle, namely the Department of Orthopaedic Surgery and Sports Medicine from the Amsterdam UMC, the Netherlands, the Department of Orthopaedic Surgery, Kyoto Shimogamo Hospital, Japan, and the Clinical and Research Institute for Foot and Ankle Surgery, Jujo Hospital, Japan. Approval by the local medical ethics committee at the Department of Orthopaedic Surgery and Sports Medicine from the Amsterdam UMC, the Netherlands was obtained prior to the start of this study (reference number: 08/326) and the study was performed in accordance with the Declaration of Helsinki.

### Patient Selection

All patients with primary MTP-1 OCLs surgically treated with arthroscopic BMS between January 2015 and August 2022 in an academic tertiary referral center specialized in, and accredited for, the treatment of cartilage injuries of the foot and ankle were potentially eligible for inclusion. All patients underwent a minimum of 3-6 months conservative therapy, and if unsuccessful, surgical treatment was considered and discussed in a shared decision-making process. Conservative therapy consisted of, or a combination of, moderation of physical activities, physical therapy, bracing, inlays, and non-steroidal anti-inflammatory medication. The operative records from 2 senior foot and ankle fellowship trained surgeons (G.K. and S.A.S.) were screened to identify potential eligible patients. The patient electronic records were independently screened by 2 reviewers (Ct.L.B. and Q.R.) to identify eligible patients by using the inclusion and exclusion criteria as shown in **Table 1**. After screening, patients were contacted by phone or e-mail to obtain consent for participation in this study and in a potential interview relating to the present study.

**Table 1.** Inclusion and Exclusion Criteria.

Inclusion Criteria	Exclusion Criteria
Symptomatic osteochondral lesion of MTP-1 joint treated with arthroscopic bone marrow stimulation	Follow-up <12 months
OCL of the MTP-1 joint confirmed by CT or MRI	Patients lost to follow-up or unwilling to participate
Failed conservative treatment for 3-6 months	End-stage osteoarthritis of the MTP-1 joint (e.g., complete destruction of the joint with joint line not recognizable on imaging)
	Polytrauma patients including open fractures to the affected joint

MTP-1 = first metatarsophalangeal; OCL = osteochondral lesion; CT = computed tomography; MRI = magnetic resonance imaging.

### Data Collection

**Figure 1.**
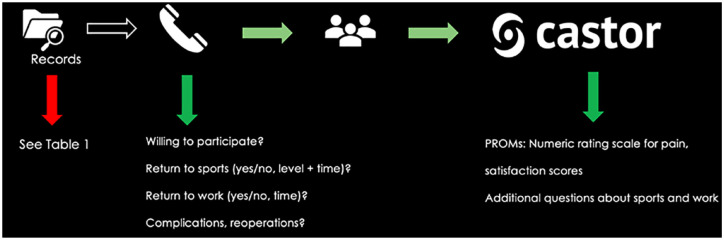
Infographic on the study process concerning data obtainment. PROMs = patient-reported outcome measures.

After obtaining informed consent, all eligible patients underwent an in-depth phone interview to collect outcomes on sports participation and return to sports, occupation before injury, return to work, and any reoperation of the lower extremities after index treatment (as depicted in **Fig. 1**, specific details on the methodology and analysis of these outcomes are described hereafter). Hereafter, patients were sent an online questionnaire through the online CASTOR© portal in order to obtain the below-described PROMs. The full protocol concerning the phone interview is available in Suppl. Appendix S1.

Patient demographics and baseline characteristics regarding patient, injury, treatment, and lesion were collected from the patient medical electronic records. Patient and injury characteristics included: age at surgery, sex, BMI, sports participation and at what level (i.e., none, recreational, competitive, or elite level), injury mechanism (i.e., traumatic or sudden onset), and concomitant injuries. Treatment characteristics included whether a debridement with or without a microfracture procedure was done, including concomitant procedures and any previous treatment concerning the MTP-1 joint.

#### Return to sports

Sports outcomes in this study were analyzed for all patients who participated in sports before the onset of the injury. Return to sports and its subcategories were defined according to a derivative of Ardern *et al.*^17^ The levels of return to sport and their definitions used for this study are shown in **Table 2**. Information regarding sports activity and return to sports was collected through the above-described in-depth telephone interview to confirm and ensure accuracy. Patients were asked in the in-depth telephone interview about sports participation and level of sport (i.e., none, recreational, competitive, or elite level) both before injury and at latest follow-up.

**Table 2.** Levels and Definitions of Return to Sports.

Level of Return to Sport	Definition of Level
Return to any level of sport	Return to any level of sport regardless of pre-injury level, with the patient being active in sports (i.e., rehabilitation, modified/restricted training, non-jumping) but not ready to be active at pre-injury level
Return to pre-injury level of sport	The return to the same level and type of sport before injury in training
Return to performance	Performing at the same level of sports in competition or even at a higher level of sports compared to the pre-injury situation

The primary outcome of this study was defined as the return to sports rate (in percentage) at any level after the surgical intervention. All other outcomes in this study were dedicated as secondary outcomes. Secondary outcomes regarding sports that were collected in this study were return to pre-injury level of sport and return to performance. In addition, return to sports times (in weeks) for the specifically associated subcategories as mentioned above and below were collected and analyzed.

#### Patient-reported outcome measures

The following PROMs were collected: the Numeric Rating Scale (NRS) for pain during different activities, satisfaction outcomes, as well as work/occupation outcomes. The NRS in rest, during walking, stair-climbing, and running was reported by the patient at the moment of follow-up. The NRS is a subjective pain scale with scores from 0 (no pain) to 10 (worst pain possible).^18^ Concerning the patient satisfaction outcomes, patients were asked to report on how satisfied they were with the following variables: the treatment that the patients received (in general/overall), the outcome after treatment at moment of follow-up, the return to sports activities, and activities of daily living at the moment of follow-up. The following subcategories were used for the degree of satisfaction: very satisfied, fairly satisfied, satisfied, unsatisfied, and very unsatisfied. In addition, patients were asked to report on their previous and current occupation (work): the patients had to report type of occupation prior to the injury, whether return to work after treatment occurred (yes/no) including the time to return to work, and what the occupation was at the latest follow-up. This outcome was verified by the in-depth telephone call.

#### Complications and reoperations

Any complications related to the surgical treatment received, reoperations, or revision surgery of the MTP-1 joint were collected from the electronic patient file(s) and verified through the phone interview. Revision surgery was defined as any surgical procedure involving treatment to the OCL of the MTP-1 joint after the index procedure. A reoperation was defined as any surgical procedure to the related joint not involving treatment to the OCL.

#### Radiological evaluation

All patients underwent a computed tomography (CT) scan and/or an MRI scan at initial presentation. In case both a CT and an MRI scan were available radiological measurements were performed using pre-treatment CT scan. The following baseline lesion characteristics were extracted: lesion size in millimeters in the anterior-posterior (AP) direction, medial-lateral (ML) direction, and depth. In addition, lesion location (i.e., metatarsal sided or phalanx sided) as well as lesion morphology (fragment/crater/cyst) were assessed.^19^

### Surgical Technique

The surgical technique as describe below is a combinatory expert opinion from both the Amsterdam experience and from the Kyoto/Chiba experience.

**Figure 2.**
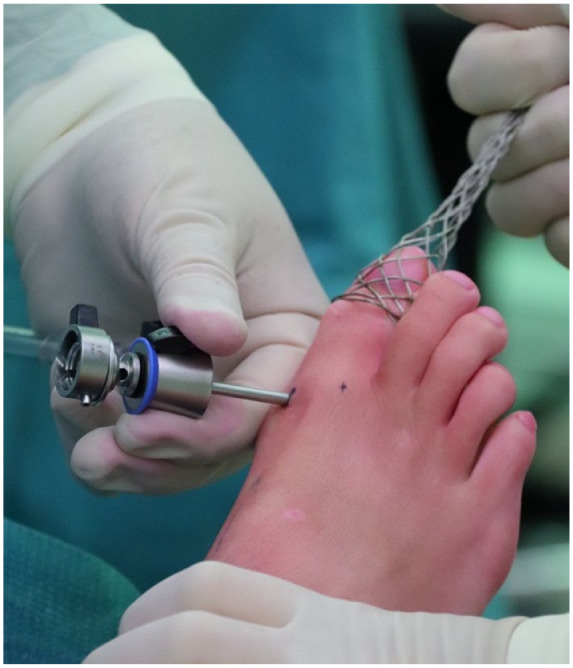
Placement of the medial portal under distraction: the dorsomedial portal is placed at the joint line and medial to the extensor hallucis longus.

The arthroscopic approach of BMS of the MTP-1 joint is performed with the patient in supine position with the heels at the edge of the operating table. A tourniquet is placed around the upper leg of the ipsilateral extremity to optimize visualization during the procedure. The patient is given a local anesthesia or general anesthesia. The affected foot and ankle are disinfected with chlorhexidine and the operative field is draped in a sterile manner. The MTP-1 joint is identified and 2 incisions are made dorsomedially and dorsolaterally of the joint. The dorsomedial portal is placed at the joint line and medial to the extensor hallucis longus (EHL) tendon, with care taken to not damage the medial branch of the superficial peroneal nerve. The dorsolateral portal is placed lateral to the EHL at the joint line (as depicted in **Fig. 2**).

**Figure 3.**
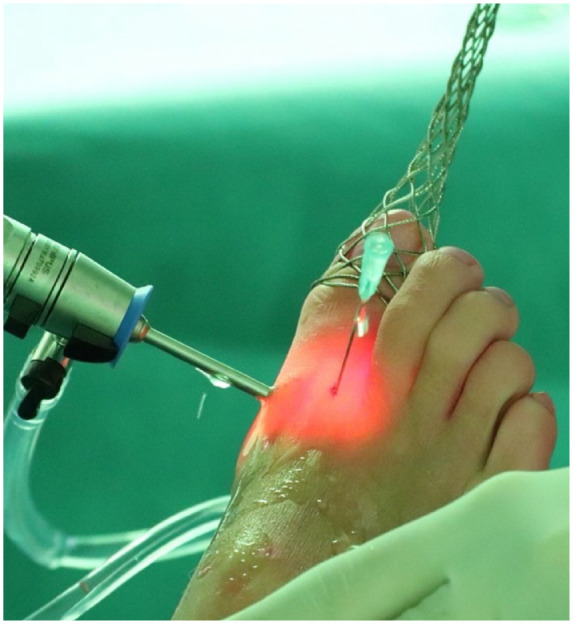
Distraction of the right first metatarsophalangeal joint and placement of the lateral portal through needle insertion (the dorsolateral portal is placed lateral to the extensor hallucis longus at the joint line).

**Figure 4.**
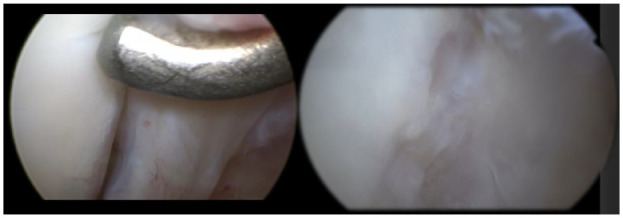
An OCL in the right first metatarsophalangeal joint located on the lateral part of the base of the phalanx. We identify and inspect the OCL with the use of a probe to identify the unstable borders of the joint. OCL = osteochondral lesion.

**Figure 5.**
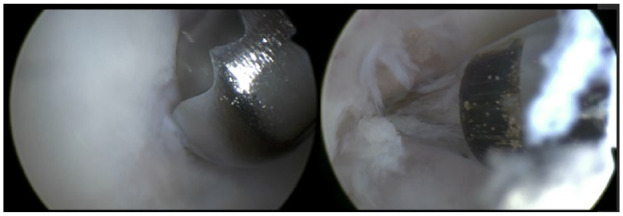
Microfracturing the osteochondral lesion (left: usage of shaver to debride unstable edges; right: a microfracture awl is used to perform the bone marrow stimulation).

**Figure 6.**
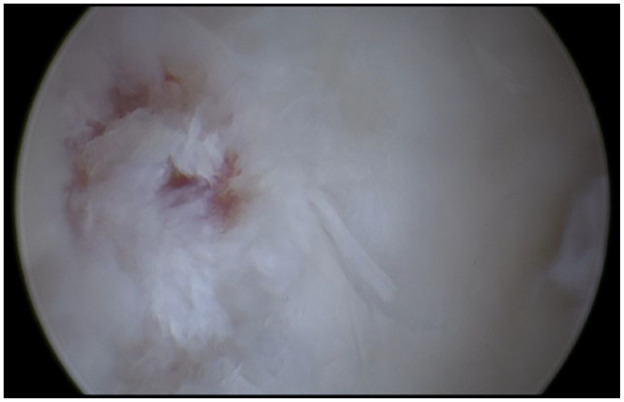
Result after microfracturing of the lesion.

A noninvasive distractor is utilized to achieve joint distraction (as depicted in **Fig. 3**). Next, a 2.7-mm arthroscope or 1.9-mm needle arthroscope, depending on surgeon preference and availability, is introduced medially and the MTP-1 joint is inspected.^20^ To optimize visualization, synovial debridement is performed as needed with a shaver. The OCL is identified and any loose or defective cartilage and sclerotic bone tissue are debrided with the shaver until a smooth surface is achieved (**Fig. 4**). A microfracture awl is additionally used to penetrate the subchondral bone until bleeding or bone marrow is visualized (**Figs. 5** and **6**). Hereafter the joint is fully inspected and thoroughly irrigated, instrumentation is removed, and the portals are sutured. Postoperatively, patients are placed in a short leg splint. The splint is changed to a Geisha boot 4-5 days postoperatively. When the swelling has reduced and the sutures have been removed 2 weeks postoperatively, the patient can start active non-weightbearing and strengthening exercises in the Geisha boot with an experienced physical therapist focused on foot and ankle rehabilitation. Patients are kept non-weightbearing until 2 weeks postoperatively. After 4 weeks postoperatively, full weightbearing will gradually be extended as tolerated. In addition, a personalized rehabilitation program focused on regaining normal gait, balance, and strength of the foot is started.

### Patient Characteristics

A total of 11 potential suitable patients were identified. Two patients had to be excluded because one patient only received conservative treatment and the other patient was lost to follow-up. In total, 9 patients were included. The overall median age was 22 (IQR: 19.5-28.5) years, and 56% of patients were males. The median follow-up time was 47 (IQR: 23-91.5) months. A full overview of the baseline patient and treatment characteristics is shown in **Table 3**.

**Table 3.** Baseline Patient Characteristics.

Patient	Patient Characteristics						Treatment Characteristics				
	Age at Surgery (Years)	Sex (M/F)	BMI (kg/m^2^)	Injury Etiology	Pre-Injury Sports	Pre-Injury Occupation	Previous Conservative Treatment	Previous Surgical Treatment	Surgical Treatment	Additional Procedures	Follow-Up (Months)
1	20	M	22.4	Traumatic	Football, competitive level	Student	Steroid injections	None	Debridement	None	94
2	19	M	25.3	Not reported	Track and field, competitive level	Full-time athlete	Restriction of physical activity, icing, and insoles	None	Debridement	None	116
3	28	F	24.8	Not reported	Judo, elite level	Full-time athlete and judo trainer	Restriction of physical activity, icing, and insoles	None	Debridement + microfracture	None	89
4	29	M	20.1	Non-traumatic/sudden onset	Dancing, elite level	Full-time athlete	Restriction of physical activity, icing, and insoles	None	Debridement + microfracture	None	47
5	20	M	20.0	Not reported	Dancing, elite level	Full-time athlete	Steroid injections	None	Debridement + microfracture	None	32
6	16	M	16.3	Non-traumatic/sudden onset	Track and field, competitive level	Student	Restriction of physical activity and insoles	None	Debridement + microfracture	None	14
7	22	F	22.8	Traumatic	Hockey, competitive level	Student	Restriction of physical activity, insoles, and walker	None	Resection lateral impingement + debridement and microfracture	None	14
8	48	F	33.1	Traumatic	None	Office job, full-time	Insoles and steroid injections	None	Debridement + microfracture	None	69
9	22	F	23.0	Traumatic	None	Student	Physical therapy and insoles	None	Debridement + microfracture	None	41
Median:	22 (IQR: 19.5-28.5)	N.A.	22.8 (IQR: 20.1-25.1)	N.A.	N.A.	N.A.	N.A.	N.A.	N.A.	N.A.	47 (IQR: 23-91.5)

BMI = body mass index; IQR = interquartile range; N.A. = not applicable; M = male; F = female.

### Radiological Lesion Characteristics

An overview of the baseline radiological lesion characteristics for each patient is provided in **Table 4**. The intraclass correlation coefficient (ICC) value for interobserver agreement was 0.94 for AP diameter, 0.94 for ML diameter, and 0.97 for depth. The Cohen Kappa value for inter-rater reliability was 0.78 for lesion location and 0.81 for the morphological classification.

**Table 4.** Baseline Radiological Characteristics.

Patient	Side	CT or MRI Scan	Lesion Location	AP Diameter (mm)	ML Diameter (mm)	Depth (mm)	Morphology
1	Right	MRI	Metatarsal, medial	3.0	5.2	7.0	Crater
2	Right	MRI	Metatarsal, medial	5.3	6.5	7.5	Cyst
3	Right	CT	Metatarsal, medial	3.1	3.4	4.0	Cyst
4	Left	CT	Metatarsal, medial	5.3	3.4	3.0	Cysts
5	Right	CT	Metatarsal, medial	6.0	4.0	3.5	Crater
6	Right	CT	Metatarsal, central	4.0	3.2	2.0	Crater
7	Right	MRI	Phalanx, central	2.6	3.4	2.0	Cyst
8	Left	MRI	Metatarsal, medial	4.5	5.2	3.9	Crater
9	Left	CT	Metatarsal, central	2.3	2.4	2.4	Fragment (non-fixable)
Median				4.0 (IQR: 2.8-5.3)	3.4 (IQR: 3.3-5.2)	3.5 (IQR: 2.2-5.5)	

CT = computed tomography; MRI = magnetic resonance imaging; AP = anterior-posterior; ML = medial-lateral; IQR = interquartile range.

### Statistical and Data Analysis

Statistical data analysis was performed with Statistical Package for Social Sciences version 26.0 (SPSS Inc., Chicago, IL). Patient characteristics, clinical outcomes, and radiological outcomes were assessed for normality. Categorical outcomes were presented as numbers and percentages. Due to the low number of patients involved in the present case series no formal statistical comparison of outcomes was made. Intra- and interobserver agreement for lesion size was assessed using the ICC according to the following methodology: Intra-rater reliability was assessed by repeating the measurements. Lesion size measurements were assessed with use of ICC analysis. ICC analysis outcomes of 0.75 to 0.90 were indicative of good reliability, and outcomes of >0.90 were indicative of excellent reliability. The Cohen kappa value was used to test inter-rater reliability for lesion location and morphological classification. Outcomes were interpreted as substantial (*k* = 0.61-0.8) or almost perfect (*k* > 0.80).^21^

## Results

### Return to Sport

Preoperatively, 7 patients (78%) participated in sports and 2 patients (22%) were not active in sports. The primary outcome, namely the return to any level of sport rate, was 86% at a median of 4 (IQR: 2.6-5.8) months postoperatively. Pre-injury, 4 patients (57%) participated at an elite level of sports and 3 patients (43%) participated at a competitive level of sports. Full details on all return to sports outcomes are presented in **Table 5**.

**Table 5.** RTS Outcomes.

Patient	Pre-Injury Sports and Level	RTS Any Level	Time to RTS Any Level (Months)	RTS Pre-Injury Level	Time to RTS Pre-Injury Level (Months)	RTP	Time to RTP (Months)
1	Football, competitive level	Yes	8	Yes	8	Yes	12
2	Track and field, elite level	Yes	4	Yes	4	Yes	8
3	Judo, elite level	No, persisting pain symptoms	N.A.	No	N.A.	No	N.A.
4	Dancing, elite level	Yes	4	Yes	4	Yes	6
5	Dancing, elite level	Yes	1, 5	Yes	1, 5	Yes	2
6	Track and field, competitive level	Yes	5	No	N.A.	No	N.A.
7	Hockey, competitive level	Yes	3	Yes	7	Yes	9
8	None	N.A.	N.A.	N.A.	N.A.	N.A.	N.A.
9	None	N.A.	N.A.	N.A.	N.A.	N.A.	N.A.
Median	N.A.	N.A.	4 (IQR: 2.6-5.8)	N.A.	4 (IQR: 2.8-7.5)	N.A.	8 (IQR: 4.0-10.5)

RTS = return to sport; RTP = return to performance; N.A. = not applicable; IQR = interquartile range.

### Patient-Reported Outcomes

The median NRS during walking at latest follow-up was 1 (IQR: 0-2.5). **Table 6** provides an overview of NRS scores per patient at latest follow-up. Regarding the return to work outcomes, all (100%) patients were able to return to their preoperative occupation without any adjustments expect for one patient who needed an adjustment in the job (Patient 3). The median return to work time was 4 (IQR: 2-17) weeks. **Table 7** provides an overview of the return to work outcomes for each patient and **Table 8** provides an overview of post-treatment satisfaction scores per patient and per outcome.

**Table 6.** Patient-Reported Outcomes: NRSs for Pain at Latest Follow-Up.

Patient	NRS in Rest	NRS during Walking	NRS during Stair-Climbing	NRS during Running
1	0	0	0	0
2	1	1	0	1
3	0	0	0	3
4	0	0	0	0
5	0	0	0	3
6	2	5	5	8
7	0	1	0	1
8	2	4	4	7
9	1	1	1	7
Median	0 (IQR: 0-1.5)	1 (IQR: 0-2.5)	0 (IQR: 0-2.5)	3 (IQR: 0.5-7)

NRS = Numeric Rating Scale; IQR = interquartile range.

**Table 7.** RTW Outcomes.

Patient	Pre-Treatment Occupation	RTW (Yes/No)	Time to RTW (Weeks)	Occupation at Latest FU
1	Student	Yes	2	Not working
2	Full-time athlete	Yes	17	Account management
3	Full-time athlete, judo trainer	Yes	4^a^	Accounting
4	Full-time athlete	Yes	17	Full-time athlete
5	Full-time athlete	Yes	6	Full-time athlete
6	Student	Yes	2	Student
7	Student	Yes	2	Student
8	Office job	Yes	26	Cleaning job
9	Student	Yes	2	Office job
Median	N.A.	N.A.	4 (IQR: 2-17)	N.A.

RTW = return to work; FU = follow-up; N.A. = not applicable; IQR = interquartile range.

a Patient was only able to return to work as a judo trainer.

**Table 8.** Patient-Reported Treatment Satisfaction Outcomes at Latest Follow-Up.

Patient	Treatment (Overall/in General)	Outcome after Treatment	Sports Activities	ADL
1	Very satisfied	Very satisfied	Very satisfied	Very satisfied
2	Very satisfied	Very satisfied	Very satisfied	Very satisfied
3	Fairly satisfied	Fairly satisfied	Fairly satisfied	Very satisfied
4	Very satisfied	Very satisfied	Very satisfied	Very satisfied
5	Very satisfied	Very satisfied	Fairly satisfied	Very satisfied
6	Unsatisfied	Satisfied	Very unsatisfied	Unsatisfied
7	Very satisfied	Very satisfied	Very satisfied	Very satisfied
8	Fairly satisfied	Fairly satisfied	Satisfied	Fairly satisfied
9	Fairly satisfied	Fairly satisfied	Satisfied	Very satisfied

ADL = activities of daily living.

### Complications and Reoperations

No complications were reported in any of the included patients. No reoperations or revision surgeries of the affected foot or MTP-1 joint after index surgery were reported in the study period.

## Discussion

The most important finding of the present study is that, in this selected cohort, arthroscopic BMS for MTP-1 joint OCLs resulted in adequate return to sports rates in athletes and non-athletes. Furthermore, good results were seen regarding the median NRS for pain during rest, walking, stair-climbing, and running, respectively. In addition, all patients were able to return to work, with most of the patients being very or fairly satisfied with the result of their treatment. There is consensus in the literature that MTP-1 joint arthroscopy is a safe and effective alternative to open techniques for the MTP-1 joint.^5,11,15,22-25^ The advantage seen is that the technique is minimally invasive, resulting in less damage to the periarticular tissues, less soft tissue dissection, smaller wounds, and less scar tissue. This results in a faster recovery and allowing patients to be active again in sports or work more quickly.^5,11,22,23^

When zooming in on the return to sports outcomes, we can state that the present study demonstrates favorable outcomes in terms of the return to sports, with nearly all patients engaged in sports prior to treatment achieving a successful return to sport outcome, and the majority of them restoring their performance to pre-injury levels. More specifically, arthroscopic BMS in patients with symptomatic OCLs of the MTP-1 joint yielded an 86% rate of return to sports at any level and a substantial 71% rate of return to pre-injury performance levels. These numbers are fairly consistent with other limited studies reporting on return to sport outcomes.^5,13-15,26^ To be precise, Debnath and colleagues^15^ reported a case series of 20 patients following arthroscopy of the MTP-1 joint with a minimal follow-up of 2 years. This series—solely including 3 patients with an OCL who underwent BMS to the MTP-1 joint—reported a return rate to former sporting activities of 90% (9 of 10 patients). The level of sport was, however, not described in this particular study and it is also unclear what the return to former sporting activities rate was of the 3 patients with an OCL. Another study, performed by Van Dyke *et al.*,^14^ reviewed the results of 9 patients that underwent particulated juvenile cartilage allograft transplantation for first metatarsal head OCLs with an average follow-up of 3.3 years. Preoperatively, all patients participated in recreational physical activity and 7 of the 9 (78%) patients were able to return to their recreational level of activity. Kim *et al.*^13^ performed a study that included a total of 22 patients with an OCL who were active in sports and underwent either subchondral drilling or osteochondral autograft transfer system treatment. Post-operatively, all patients returned to recreative sports activities—the patients in the subchondral drilling group did not return to sports activities at an earlier state (both 16 weeks postoperatively) than the osteochondral autograft transfer system treatment. As such, we can conclude that when considering the current literature,^5,13-15,26^ it is challenging to make a direct comparison with the current study. This, is because the athlete population in these studies is predominantly active at the recreational level as opposed to the competitive and elite level of the athletes included in our study population. Furthermore, in the studies as mentioned above and as included in the literature, the level of sports is in the majority of the cases not mentioned, and it should be stated that solely a proportion of the included patients in the abovementioned cohort included patients with an OCL to the MTP-1 joint.

In the literature, favorable to excellent clinical outcomes, ranging from 80% to 100%, have been documented for the treatment of OCLs in the MTP-1 joint.^5,11,13,15,26-32^ Ferkel^25^ conducted a study involving 22 patients who underwent MTP-1 joint arthroscopy, including 3 patients with OCLs. The results demonstrated good to excellent clinical outcomes in all OCL cases. Similarly, Debnath *et al.*^15^ reported on a series of 20 patients with intra-articular problems to the MTP-1 joint, with a minimum 2-year follow-up, wherein 3 patients presented with OCLs. Comparing preoperative and postoperative American Orthopaedic Foot and Ankle Society (AOFAS) scores, the study showed good to excellent outcomes, with a mean improvement from 10 to 100. Notably, all patients achieved a fully functional and pain-free MTP-1 joint. In a study by van Dijk and colleagues,^5^ 4 patients with OCLs were examined in a series of 24 patients, with a minimum follow-up duration of 2 years. The assessment utilized a non-validated (i.e., subjective) scoring system, and 3 out of 4 patients exhibited good to excellent results. Davies and Saxby^27^ studied 11 patients with OCLs, including 5 patients, with a mean follow-up of 19.3 months. At final follow-up, all patients reported excellent results concerning pain relief, although 3 patients mentioned residual stiffness. In the study conducted by Kuyucu *et al.*,^32^ 14 patients with OCLs were monitored for a mean duration of 16.4 months. Postoperatively, all patients experienced improved AOFAS scores, increasing from 48 preoperatively to 87, alongside decreased Visual Analog Scale (VAS) pain scores from 7-10 to 1-3 at final follow-up. Similarly, Kim *et al.*^13^ observed 13 patients in a consecutive series of 22 patients, with a mean follow-up of 25.1 months. The mean VAS score significantly improved from 6.9 to 3.9, and patients reported good to excellent outcomes. These results are in concordance with the good to excellent clinical outcomes observed in the present study. As for patient satisfaction, Ahn *et al.*^11^ reported on a consecutive series of 59 patients, with a mean follow-up of 25 months, showing that 95% of patients were satisfied with the procedure at final follow-up. Similarly, in the present study, 5 patients expressed being very satisfied, and 3 patients reported being fairly satisfied.

This study needs to be interpreted in light of its strengths and limitations. First, a small number of patients was included in this retrospective study. Given the limited data available and heterogeneity in the study group, care must be taken when drawing firm conclusions. A strength of this study is the high follow-up rate and the high response rate of the approached patients. In addition, an extensive in-depth dynamic qualitative interview regarding return to sport was conducted which improved the quality assurance of the accuracy of the return to sport outcomes. Moreover, this was the first and only study focusing on specific sports–level outcomes in OCLs of the first MTP joint, including return to performance levels. Other strengths include the use of a second rater for extracting data and performing the measurements of lesion size in all directions.

A scarcity of literature exists concerning sports outcomes after treatment of OCLs located in the MTP-1 joint, despite the potential value of return to sports rates as a decisive factor in clinical decision-making. Effectively managing patient expectations is of utmost clinical importance in shared decision-making, and therefore, the findings from this study hold significant clinical relevance. The outcomes of this investigation offer valuable insights for both physicians and patients, aiding them in the collaborative decision-making process. Looking ahead, further studies encompassing a larger cohort of patients and extended follow-up periods are imperative to validate and reinforce the clinical and sports-related outcomes of MTP-1 joint interventions. Such research endeavors will better equip healthcare professionals and patients with comprehensive data, ultimately facilitating more informed treatment choices and improved mid- to long-term prognosis.

## Conclusion

Arthroscopic BMS for patients with symptomatic OCLs to the MTP-1 joint can be considered safe and yields an 86% return to sport at any level and a 71% return to pre-injury and performance level, with good clinical, return to work, as well as satisfaction outcomes.

Furthermore, all patients were able to return to work postoperatively and 8 out of 9 patients were fairly to very satisfied with the results of the treatment.

## Supplemental Material

sj-docx-1-car-10.1177_19476035231200332 – Supplemental material for Back in Action: High Return to Pre-Injury Level of Sports after Arthroscopic Bone Marrow Stimulation for Osteochondral Lesions of the First Metatarsophalangeal (MTP-1) Joint Supplemental material, sj-docx-1-car-10.1177_19476035231200332 for Back in Action: High Return to Pre-Injury Level of Sports after Arthroscopic Bone Marrow Stimulation for Osteochondral Lesions of the First Metatarsophalangeal (MTP-1) Joint by Carlijn S. ter Laak Bolk, Quinten G.H. Rikken, Jari Dahmen, Yoshiharu Shimozono, Masato Takao, Sjoerd A.S. Stufkens and Gino M.M.J. Kerkhoffs in CARTILAGE
