# Supplementary material for: Back in Action: High Return to Pre-Injury Level of Sports after Arthroscopic Bone Marrow Stimulation for Osteochondral Lesions of the First Metatarsophalangeal (MTP-1) Joint
Source: Cartilage. 2023 Sep 21;15(1):47–57. doi: 10.1177/19476035231200332 (PMC10985395; doi:10.1177/19476035231200332)
Supplement: sj-docx-1-car-10.1177_19476035231200332 – Supplemental material for Back in Action: High Return to Pre-Injury Level of Sports after Arthroscopic Bone Marrow Stimulation for Osteochondral Lesions of the First Metatarsophalangeal (MTP-1) Joint [file sj-docx-1-car-10.1177_19476035231200332.docx]

**Appendix 1**

Full protocol used for the phone interview in this interview

First a few quick questions:

- Did you have surgery on the affected foot/ankle after the index operation? If so, what hospital was this in and what kind of surgery?
- Did you have any complications after the treatment you received?

**Pre-injury characteristics**

*Sports*

- Were you active in sports before injury?
- If so, which sport was this and at what level did you participate before your injury?
  - Top level/Professional (OS, World Cup, European Championship, World Cup, National team, Dutch National Championships, paid sport, team competition honorary/1st division)
  - Competitive (football/hockey league lower than 1st division)
  - Recreational (more than 50 hours per year in sports)
- How many hours per week did you exercise before injury and treatment?
- Were you active in any other sport/activity before treatment?

*Work*

- Did you work before your injury/treatment?
- If so,
  - What kind of work did you do before the injury/treatment?
  - How many hours per week did you work before the injury/treatment?
  - Did you do any other type of work (i.e., second job) before your injury/treatment?

**Post-treatment characteristics**

*Return to sports*

- Were you able to return to sports after your operative treatment?
- When was this?
- To which sport were you able to return?
- How many hours per week did you exercise after treatment?
- At what level were you able to return to sports?
  - Top level/Professional (OS, World Cup, European Championship, World Cup, National team, Dutch National Championships, paid sport, team competition honorary/1st division)
  - Competitive (football/hockey league lower than 1st division)
  - Recreational (more than 50 hours per year in sports)
- Were you able to return to the same level of sports in training compared to pre-injury level?
  - If so, when?
- Were you able to return to the same level of sports in competition compared to pre-injury level?
  - If so, when?
- Were you able to return to a higher level of sports after treatment compared to pre-injury level?
  - If so, when?
- Do you currently (still) participate in sports?
- Which sport do you currently participate in?
- How many hours per week do you exercise?
- At what level are you currently active?
  - Top level/Professional (OS, World Cup, European Championship, World Cup, National team, Dutch National Championships, paid sport, team competition honorary/1st division)
  - Competitive (football/hockey league lower than 1st division)
  - Recreational (more than 50 hours per year in sports)

*Return to work*

- Were you able to work after your treatment?
- When was this?
- To what type of work were you able to return?
- How many hours per week did you work after treatment?
- Were you able to do the same type of work and/or number of hours after the injury/treatment as before the injury/treatment?
  - If not, may I ask why not?
- Are you currently (still) working?
- What type of work?
- How many hours per week do you work?
- Are you currently doing the same type of work after injury/treatment as before injury/treatment?
  - If not, may I ask why not?
